# Effects of Cognitive Behavioral Treatment-Based Withdrawal Intervention in Patients with Long-Term Opioid Use for Chronic Pain

**DOI:** 10.3390/jcm14217640

**Published:** 2025-10-28

**Authors:** C. Paul van Wilgen

**Affiliations:** 1 Transcare Transdisciplinary Pain Treatment Center, 9722 Groningen, The Netherlands; p.vanwilgen@transcare.nl; 2 Pain in Motion Research Group (PAIN), Department of Physiotherapy, Human Physiology and Anatomy, Faculty of Physical Education & Physiotherapy, Vrije Universiteit Brussel, 1090 Brussels, Belgium

**Keywords:** opioid withdrawal, central sensitisation, pain education, CBT chronic pain

## Abstract

**Objectives**: Prolonged opioid use leads to tolerance and hyperalgesia in patients with chronic pain. Apart from an increase in pain, opioid use also leads to several other adverse effects. Nevertheless, the prevalence of opioid use as a treatment for chronic pain remains high, and opioid withdrawal interventions deserve more attention. This study evaluates the effects of a guideline for an opioid withdrawal intervention method that is nested in cognitive behavioral treatment (CBT) and is specifically for patients with a history of long-term opioid use and chronic pain. **Methods**: We conducted a clinical, exploratory, and mixed-methods study involving pre- and post-measurements on opioid use and health-related quality of life (SF-36), as well as a qualitative analysis of patient experiences (interviews) to evaluate the program. **Results**: A total of 29 patients were included in the study; 23 of these patients no longer used opioids, and some continued withdrawal under the guidance of their general practitioner. Quality of life improved in all domains, including the amount of pain experienced. No patients reported increased pain levels, and most experienced significantly fewer adverse side effects. Patient satisfaction was high, with no negative long-term side effects of the intervention reported. **Conclusions**: In light of the results of this study, it is important to address opioid use in patients with chronic pain. There are strong arguments in favor of motivating patients to withdraw from using opioids to treat chronic pain, which can be achieved in combination with CBT.

## 1. Introduction

Chronic pain is pain that persists or recurs for longer than 3 months, with pain becoming the predominant clinical problem. Chronic pain is a frequent condition, affecting an estimated 20% of people worldwide, and is always a multifactorial condition in which biological, psychological, and social factors contribute [1]. Chronic pain is explained by neurophysiological changes, commonly referred to as nociplastic pain, with central sensitization as its underlying mechanism [2]. It has long been known that prolonged opioid use leads to increased tolerance and heightened hyperalgesia based on central sensitization [3]. Neuroplastic changes in the peripheral and central nervous system lead to sensitization of several pain pathways, including top-down inhibiting or facilitating pathways [4,5]. So opioid-induced hyperalgesia contributes to the increase in pain over time in long-term users. This increase in pain often leads to an increase in opioid use, leading to more adverse side effects, physical dependence, and, therefore, a negative quality of life in all domains [6]. Any physically experienced negative side effects can be several and severe, such as gastrointestinal complaints, excessive sweating, itching, sleep disordered breathing, sexual dysfunction, fatigue, mood disorders (depression), low levels of sex hormones, higher risk of fractures, “brain fog,” heart attacks, and dental problems [7].

This increase in chronic pain is also seen in the population treated with opioid substitution therapy, primarily for opioid use disorder (OUD). The prevalence obtained in a meta-analysis was 45.3% (95% CI [38.7–52.1]), and this prevalence is more than twice as high as the prevalence of chronic pain in the overall population [5]. This finding seems to underline the increased pain sensitivities of long-term opioid users. Problematic opioid misuse is therefore described in the *Diagnostic and Statistical Manual of Mental Disorders, Fifth Edition (DSM-5)* as opioid use disorder (OUD). To prevent patients from developing OUD, several international guidelines advise against the prescription of opioids for chronic pain. Prescribing opioids puts patients in a complex situation. They initially experience a reduction in pain due to the opioid, but within a few months, a paradoxical pain-enhancing effect may occur, which in many cases can lead to dose adjustments that only provide temporary relief and result in hyperalgesia in the long term. This psychologically complex learning situation of short-term pain reduction will lead to—despite long-term hyperalgesia and an increase in pain—the belief that opioids alleviate pain. Therefore, opioids are particularly unsuitable and addictive for the treatment of chronic pain. Despite this, the use of opioids has increased in recent decades, primarily for patients with chronic pain, both in the United States and in Europe [8]. Several risk factors have been identified for newly persistent opioid use. Opioids have become a routine component of post-operative pain management in recent decades, mainly due to being excessively prescribed by surgeons [9]. This post-operative overprescribing of opioids is an important risk factor for persistent opioid use and long-term adverse side effects [10]. Prescribing opioids pre-operatively is a risk factor for prolonged opioid use after surgery, independent of the type of surgical procedure [11]. Opioid use before surgery is also a predictor of increased risk for a higher average daily oral use after surgery [12]. Apart from opioid use, both an increased body mass index and the existence of three or more comorbid pain sites are risk factors before surgery. Notably, these increased pain sites (widespread pain) can be related to central sensitization. The existence of central sensitization before surgery can lead to difficulties in post-operative pain reduction [12]. Another factor that has led to an increase in opioid use in the last decade is the complex interplay between transnational pharmaceutical companies and global health systems, pain advocacy groups, pain experts, prescribers, and public health, with each group influencing opioid promotion [13]. Advocates of medical (prescription) opioid use perpetuate the concept that pain is the fifth vital sign, the incorrect belief that opioid addiction is rare, and the incorrect perception that acute pain should be treated medically to prevent chronic pain [14]. Another factor contributing to medically prescribed opioid use is the lack of knowledge about chronic pain and central sensitization among healthcare professionals [15]. The recognition of central sensitization is important in determining which pain management strategies are designated, with or without opioids. Another factor might be the bidirectional prevalence of mental health diagnoses, which is prevalent in opioid users. The role of mental health factors in chronic pain and central sensitization is widely known. Specifically, opioid users within the group of chronic pain patients are a particularly mentally vulnerable group, and this should be followed up closely. In particular, comorbid PTSD is often complex and inextricably intertwined with opioid use. This also shows that besides medical care, mental care is of importance, especially during withdrawal [16,17].

Fortunately, there is growing awareness among healthcare providers that long-term opioid use is undesirable. However, opioid withdrawal is not easy for both patients and prescribers. Despite their often severe complaints, patients believe and have learned that they need opioids for pain management, often because they initially experienced relief when starting or increasing their dosage. Pain is still perceived as the result of physical damage, necessitating opioids to suppress it. Patients may therefore be apprehensive about making any changes to their pain management strategy and may be fearful of withdrawal symptoms, i.e., increased pain. When discussing the (dis)use of opioids, patients often experience feelings of injustice or anger that opioids were prescribed or may feel like their doctor is abandoning them. Prescribers, on the other hand, are often uncertain about withdrawal and how to respond to the concerns of patients, and whether they can manage the withdrawal and how to support patients [18].

Withdrawal will lead to opioid withdrawal symptoms (OWSs); several symptoms are described, including pain, but also muscle spasms, tremors, abdominal cramps, nausea, diarrhea, anxiety or feelings of depression, restlessness, irritability, insomnia, chills, sweating, pupillary dilatation, and yawning. The severity and duration of OWSs vary with the duration of opioid use and patient-specific characteristics, including health and mental status. The distress and pain in the first days can be severe [19]. In 57% of patients with chronic pain and opioid use, OWSs were reported as the primary reason for continuing the use of opioids [20]. The question is, how can we motivate patients to change these perceptions and withdraw from long-term opioid use, and what are the effects of doing so? Withdrawal interventions often have a pharmacological approach, in which OWSs are either medically treated [21,22] or combined with psychological treatments [23,24]. For both interventions, further research is needed to determine the effectiveness of dose reduction and long-term relapse prevention. Therefore, in 2021, we developed a guideline within a transdisciplinary mental healthcare setting that aimed to promote the withdrawal of opioids in chronic pain patients, using cognitive behavioral treatment (CBT) methods.

The objective of the study, therefore, was to examine the effects of a transdisciplinary intervention on opioid withdrawal, based on a cognitive behavioral program for chronic pain, to assess the effect on opioid use, health-related quality of life, and patient experiences.

## 2. Materials and Methods

### 2.1. Participants and Setting

Patients were referred by their general practitioners or medical specialists to Transcare, a transdisciplinary mental healthcare clinic. All patients were referred to the clinic for treatment for somatic symptom disorder (SSD) or chronic pain with or without a specific request for opioid withdrawal. According to the DSM-5, a somatic symptom disorder (SSD) is characterized by one or more distressing somatic (physical) symptoms that lead to significant disruptions in daily life. The disorder is further defined by excessive and disproportionate thoughts, feelings, and behaviors related to these symptoms. The physical symptoms themselves cannot be linked to an underlying medical condition. The existence of pain without an underlying medical condition is explained by neurophysiological changes [2].

Included in the study were patients diagnosed with SSD that were older than the age of 18 and had a history of opioid use lasting more than 3 months. Exclusion criteria for the treatment included severe psychological problems for which ambulant opioid withdrawal was not suitable, dependence on other substances that could interfere with opioid use withdrawal, and a lack of social support.

### 2.2. Guidelines

All patients received a matched care intake, which included a one-hour intake by a physician/addiction specialist and a one-hour intake by a psychologist/nurse specialist. Within the transdisciplinary framework, both healthcare providers used the PSCEGS model [25]. This integrated diagnostic process first identified the underlying pain mechanism (nociceptive, neuropathic, central sensitization, or a combination); furthermore, somatic, cognitive, emotional, behavioral, and social factors related to a patient’s chronic pain were also identified. In the somatic assessment, the medication was analyzed. Specifically, patients’ motivation or willingness to undergo withdrawal and their knowledge of opioids were assessed in the cognitive section. After the assessment, CBT for chronic pain starts with pain education [26]. CBT is a psychological therapy that aims to manage pain by changing unhelpful and negative thoughts, thinking patterns, and behaviors through the development of effective coping strategies. Pain education includes explaining the difference between acute pain (“damage model”) and chronic pain (“central sensitization”). In pain education, the neurophysiology of acute and chronic pain is explained and supported by the use of metaphors. The metaphor of a “medicine cabinet” was often used in case of opioid misuse: “Taking opioids for more than 3 months shuts down your body’s own pain-relief system, leading to more pain and more need for opioids.” Pain education was delivered in two interactive sessions lasting 45–60 min. The first one was provided by the physician/addiction specialist, and the second session by the psychologist/nurse specialist. Education focuses on the explanation of sensitization and the effect of opioids in this process. Most of all, pain education is important to build an integrated, shared model of biopsychosocial factors that influence the process of chronic pain. Importantly, the different side effects of opioids were discussed. Most patients are not aware that many of their complaints are about the side effects of opioid use. In pain education, the use of motivational interviewing techniques is essential for motivational change [27]. One of the goals was to change the perception of the role of opioids from a helpful drug to a drug that aggravates pain and causes several adverse side effects. After the second education session, patients had to set their personal goals; a goal for inclusion in this intervention was to withdraw from opioids. Apart from withdrawal, additional goals for treatment were set.

In step three, a treatment plan was developed through shared decision-making. Various methods were used to withdraw from opioids. Withdrawal symptoms such as pain and other symptoms were monitored, and patients received psychological support during this process. To prepare patients for withdrawal, opioid withdrawal symptoms were explained. The importance of understanding these symptoms was emphasized. Treatment included a psychological CBT intervention, supplemented with behavioral interventions such as graded activity, exposure, and ACT, which are typically offered during CBT for chronic pain.

The withdrawal started with stabilizing the doses (time-contingent intake), and immediate release (i.r.) opioids were gradually converted to controlled release (c.r.) opioids. In some cases, opioids were switched at some point to alternatives like methadone or buprenorphine, or from pills to drops in the case of tramadol. This approach varied per patient, and the procedure was guided by an addiction physician, physician, or nurse specialist. No other pain medication was prescribed during the process, although patients were free to take medication (i.e., NSAIDs, PCM) that they were already using. In some patients, other medication was temporarily used during the withdrawal period, such as sleep medication, SSRIs or clonidine.

Withdrawal was part of the shared decision-making process; steps toward reduction were made together with the patient. Increased pain was not medically treated during withdrawal, but as in chronic pain treatments, there was a focus on changing how patients perceive and cope with pain. Before the withdrawal intervention, the patient’s general practitioner, pharmacist, and family members were informed and involved. The guidelines contained a hybrid program; intake and education sessions were face-to-face, but the majority of the withdrawal program was offered online or by telephone.

### 2.3. Measures

In this exploratory clinical mixed-method study, measurements were taken before and after treatment. These included demographics on age, gender, duration of opioid use, opioid usage, other pain medication, and RAND-36 (Dutch language version), a widely used questionnaire measuring perceived health and quality of life. Domains included general health, health changes, social functioning, emotional and physical role limitations, mental health, vitality, and pain, along with a patient satisfaction score. Each domain consists of multiple items, and each item has a specific response scale. Each item response is recoded so that higher scores consistently reflect better health. For each domain, the recoded item scores are summed. The average raw score is transformed from 0% (worst possible health status) to 100% (best possible health status) [28]. After treatment, a brief qualitative interview was conducted by telephone by the author (PvW) to analyze the personal experiences during withdrawal and the effects post-treatment. The experiences were presented, and no further analyses were conducted.

### 2.4. Ethical Considerations

This study was registered and approved by the medical ethical commission before the initiation of the study (METc nummer: 202200318). Informed consent for participation was obtained from all subjects involved in the study.

## 3. Results

In 2021 and 2022, 32 individuals were included. During the program, three patients dropped out, one patient was referred to a clinical program focusing on addiction care, and two patients decided not to proceed with the treatment after the intake and education sessions. Data from 29 patients were included in the study: 16 women and 13 men, with an average age of 49.9 years (SD 13 years). The average duration of opioid use was 4.8 years (SD 4.0 years, minimum 1 year, maximum 18 years). Dosages before and after treatment are described in Table S1.

Of the 29 patients, 23 no longer used opioids after withdrawal. One patient developed another condition during the intervention, which led to discontinuation of the withdrawal attempt. One patient continued taking tramadol due to the development of a physical condition. Two patients decided to continue withdrawal in collaboration with their general practitioner during the intervention.

The topics of the interview after the program are presented in Table 1.

Patients described the withdrawal process as difficult, with significant pain and symptoms such as fatigue, sleep disturbances, restless legs, gastrointestinal problems, vivid dreams, the exacerbation of PTSD symptoms, and itching. Two patients experienced PTSD dysregulation, requiring treatment. None of the patients experienced relapses or other severe side effects during or after withdrawal. Twelve patients reported no longer using any pain medication at all. None of the patients reported increased pain after withdrawal. Of the reported group, 24 (82%) completed the RAND-36 before treatment, and 16 (55%) completed it after treatment. (see Table 2). Improvements were observed across all domains, including health changes, physical, social, and emotional functioning. A significant improvement in the pain domain was also noted. There was a high satisfaction score.

In the long term, none of the patients reported more pain after the intervention. Some found it challenging that the pain did not disappear, but everyone stated that the withdrawal was essential to them.

## 4. Discussion

This exploratory and transdisciplinary clinical study set within an outpatient specialized mental healthcare setting described the results of guidelines for the withdrawal of opioids combined with the use of CBT to treat pain in patients with SSD and chronic pain. This personalized integrated intervention showed indications that the treatment may be an effective and feasible method for opioid withdrawal in patients with chronic pain. 

Several clinical guidelines discourage opioid use for the treatment of chronic pain. Many of these guidelines, however, give minimal advice on how to discuss this with patients who are already using opioids. A qualitative study of Henry et al. (2019) described three themes to manage this in opioid users [29]. Firstly, they found that it is essential to take note of dynamic changes in patients’ social relationships, emotional states, and health statuses, because their pain levels and perceived need for opioids can fluctuate daily [29]. This was one of the key elements of our intervention, in order to offer personalized interventions that strongly integrate the psychosocial factors of chronic pain and not only focusing on medical issues or medication to deal with fluctuations. Furthermore, instead of performing standardized predefined tapering, withdrawal should be based on shared decision-making and individualized intervention. In our intervention, a patient can decide when to make the next withdrawal step and when to lower the dose during the day. Another aspect of the guidelines and one of its themes was to address the social and emotional dynamics that are likely to impact patients’ withdrawal; for instance, addressing patient fears, focusing on patients’ best interests, and providing anticipatory guidance about withdrawal. In our guidelines, psychosocial support and pain education were key elements during the withdrawal period. In chronic pain and central sensitization, the spreading of pain is a key feature. During opioid withdrawal, unexpected “new” pain sides were common. New sides can be threatening and frightening for patients, and therefore the explanation of pain as a neurophysiological process of sensitization and the normalization of these new pain sides is important to reduce fear, and to encourage patients to take another step in the withdrawal process.

Despite the common and severe withdrawal symptoms we observed during the program, no long-term negative effects from withdrawal were reported. During the program, patients reported exacerbations in PTSD symptoms; for instance, nightmares or anxiety-induced dreams combined with sleeping problems or emotional distress. Comorbid PTSD and OUD are often complex and inextricably intertwined, since opioids seem to alleviate PTSD symptoms. In the screening and assessment period before the program, trauma screening was therefore incorporated to pre-screen for risks of PTSD exacerbation. In our integrated program, we could offer parallel PTSD interventions alongside CBT if needed. These exacerbations can also be a reason for a pause in the withdrawal. Along with an experienced healthcare team, the role of social support is of great value in ambulant programs. The involvement of well-organized experts with experience can play a significant role in the motivation of patients during withdrawal programs.

Despite the growing knowledge about the negative effects of opioids for chronic pain management and the positive impacts of withdrawal, the discontinuation of opioids remains an emotionally distressing experience for most patients, many of which experience feelings of neglect, anger, helplessness, and unhappiness, as described by Hao et al. (2014) [30]. Even so, the majority of patients with long-term opioid use assumed that their withdrawal from opioids would lead to a loss of independence in daily activities, an inability to enjoy their lives, and an inability to work [30]. In addition to feelings of perceived injustice, most users expressed the message that the prescription of opioids is not helpful and should be discontinued. This could lead to an individual’s awareness that the use of medication made their situation worse. This appraisal could lead to feelings of unfairness, a sense of loss, or that something cannot be rectified [31]. All these emotional factors and feelings of injustice should be recognized and addressed before starting a withdrawal program.

To make the transition from a “I need this” mentality to a one of “opioids aggravate my pain” is a complex process. This will require an explanation on chronic pain and the role of opioids, patients’ trust in healthcare providers, room for psychosocial interventions, and a personal withdrawal plan with regular consultations. It seems valuable for general practitioners to learn how to deal with these problems and how to educate long-term users. Above all, prevention of unnecessary opioid use in acute care settings (e.g., hospitals), is most important. It is assumed that effective pain management in the acute phase prevents chronic pain; however, this assumption is not supported by the literature. It might be prudent to reconsider opioid prescribing practices to reduce the need for withdrawal programs and patient suffering. When dealing with chronic pain, learning about pain, undergoing an integrated chronic pain analysis, and using new coping strategies could lead to high benefits.

Our guidelines describe an outpatient intervention that is provided in a hybrid setting. The first steps, assessment and education, are based on a personal setting. We believe that a patient’s trust in healthcare professionals, and their competence is crucial in the patient–healthcare professional relationship, which again is the basis of a successful treatment plan. After setting clear goals, the withdrawal intervention and CBT can be continued online or through telephone-based consultations. The content of CBT is not standard because of periods of heavy withdrawal signs, so the intervention is adapted to the situation. This also accounts for the behavioral aspects like graded activity, which is offered between the steps of withdrawal and also after the completed discontinuation of opioid use.

There are several limitations to this study. First, we conducted straightforward pre- and post-measurements in clinical practice. Methodologically stronger studies are needed to confirm our findings. Not all patients could be motivated to participate in the study during and after the intervention; it is difficult to determine the impact on the results. Some of the reasons for their unwillingness to fill out questionnaires were specific, such as a lack of interest and language barriers, but some reasons were unknown. We do believe that perceived injustice, the feeling of being neglected in healthcare settings, and anger might have interfered with the motivation to participate in the study [30]. The dropout rate for the intervention was only 10%, which is low. It is well known that dropout rates are around 30% in substance use withdrawal studies [32] This study did not provide insights into long-term outcomes, such as relapse, dysregulation, or increased pain over time. We did not check on treatment fidelity of the guideline. So, with the small sample size in this study, it is insufficient to demonstrate the effectiveness of CBT in this patient group. So, our results should be interpreted with restraint, and the guidelines should be investigated in different settings. An advantage is that this is an outpatient intervention, which can be cost-effective compared to clinical interventions. In the study, we focused on opioids, but it should be mentioned that in several patients, other medications were also part of the withdrawal program, and we could not determine the effects of this.

## 5. Conclusions

This study demonstrates that an integrated and personalized withdrawal intervention for opioid use for chronic pain based on CBT seems to have beneficial effects on pain, reduces negative side effects, and improves quality of life. It shows high satisfaction levels from the participants, and this outpatient program can be cost-effective compared to inpatient programs.

## Figures and Tables

**Table 1:** Experiences of patients and their perceived outcomes of the opioid withdrawal intervention.

	Quote
**Negative experience on opioid use**	Q: (man, age 52) “It’s worthless stuff, it should be taken off the market. I’m a much better now, and I’m glad the damage from oxycodone isn’t permanent.” Q: (woman, age 71) “I don’t want to go back to opioids anymore”.
**Positive change on symptoms**	Q: (man, age 52) “Sweating is gone; everything was too much for me before. My mood is much better; I’m back in the present (I was out of it before)”. Q: (woman, age 61) “I had a lot of side effects, nausea is gone, I can smell normally again, pain has remained the same, concentration is much better.” Q: (woman, age 49) “Better mood, better mobility, better concentration—for example, I can read again—more active, better sleep.”
**Overall change**	Q: (man, age 30) “I can do much more, my digestion is working again, no longer need Movicolon sachets.” Q: (women, age 71) “Hobbies are going better, less drowsy, I feel much better without them, I was very confused with the patches, and I don’t want them anymore.” Q: (man, age 49) “much better energy, back to work, came out of a kind of fog, much better quality of life.” Q: (man, age 39) “sleep is better, dependency is gone, emotionally more stable, better mood, less irritable, significantly better.” Q: (man, age 51) “Things are currently bad, lots of pain complaints, lower back strain and nerve entrapments. Deliberately chose not to take medication (opiates), the pain is better”.
**Change in pain**	Q: (man, age 30) “Pain is unchanged” Q: (woman, age 71) “Less pain from burns”. Q: (man, age 49) “Same pain experience with and without opioids” Q: (man, age 39) “Much fewer pain peaks” Q: (man, age 52) “Six months after stopping, after eight years—pain-free!” Q: (man, age 38) “Much less pain, much better mood!”
**Negative outcomes**	Patients reported various withdrawal symptoms during the tapering period, and the most important effect was an increase in pain but also vivid dreams, restlessness, night sweats, fatigue, and bad sleep. No long-term negative outcomes were reported from a physical perspective.

**Table 2:** Outcomes for the domains of the RAND-36 before and after the intervention, thereby added the satisfaction score with the results of the treatment.

	Before Treatment (n = 24)	After Treatment (n = 16)
**SF36 General health**	36.75%	36.88%
**SF36 Health changes**	41.07%	75.00%
**SF36 Physical functioning**	35.00%	45.94%
**SF36 Social functioning**	41.45%	61.72%
**SF36 Role limitations due to physical problems**	50.00%	82.14%
**SF36 Role limitations due to emotional problems**	76.19%	90.00%
**SF36 Mental health**	58.00%	65.75%
**SF36 Vitality**	34.75%	43.75%
**SF36 Pain**	26.17%	42.59%
**Satisfaction score with results of treatment (0–10)**	n.a.	8.83

For each domain of the RAND 36, the average raw score is transformed to a 0% (worst possible health status) to 100% (best possible health status). n.a.—not applicable.

## Data Availability

Data of our patients/participants is not available due to privacy reasons. All other data is within the article.

## Supplementary Materials

The following supporting information can be downloaded at https://www.mdpi.com/article/10.3390/jcm14217640/s1, Table S1. Pain medication, including opioids, used before and after the intervention.
