# Supplementary material for: Effects of Cognitive Behavioral Treatment-Based Withdrawal Intervention in Patients with Long-Term Opioid Use for Chronic Pain
Source: J Clin Med. 2025 Oct 28;14(21):7640. doi: 10.3390/jcm14217640 (PMC12608561; doi:10.3390/jcm14217640)
Supplement: Supplementary file 1 [file jcm-14-07640-s001.zip › jcm-3870260-supplementary.pdf]

Tabel S1: Pain medication including opioids before and after intervention

Nr	Pain medication before intervention	Pain medication after intervention
1	Buprenorphine 20 mcg<br>Codeine 10 mg 8 p.d.<br>Pregabalin 75 mg 1 p.d.<br>Paracetamol 1000 mg 4 p.d.	Pregabalin 75 mg 1 p.d.<br>Paracetamol 1000 mg 4 p.d.
2	Fentanyl patch 100 ug 2 pieces<br>Gabapentin 600 mg 6 p.d<br>Oxycodone 5 mg 6-10 p.d.<br>Paracetamol 500 mg 6 p.d.	Paracetamol 500 mg 6 p.d.
3	Oxycodone tablet c.r. 10 mg 5 p.d<br>Diclofenac 50mg 1p.d 1	No pain medication
4	Oxycodone tablet c.r. 5 mg 4 p.d.<br>Oxycodone capsule i.r. 5 mg 1-6 p.d.<br>Pregabalin caps 75 m.g 5 p.d.	Paracetamol 500mg 2 x 2 p.d.<br>Pregabalin caps 75 mg 5 p.d.
5	Oxycodone Tablet 10 c.r. mg. 3 p.d	Methadone, 8 mg p.d.
6	Tramadol 50 mg 3 p.d.	No pain medication
7	Oxycodone caps 5 mg 2-6 p.d<br>Fentanyl patch 12 ug<br>Amitriptyline 25 mg, 2 p.d<br>Paracetamol 1000 mg 4 p.d.	Paracetamol 1000 mg 4 p.d.
8	Tramadol 2 x 50 mg 2 p.d.	No pain medication
9	Oxycodone 5 mg 3 p.d.<br>Pregabalin 75 mg 3 p.d<br>Now and then Paracetamol	Now and then Paracetamol
10	Naproxen tabl. 250 mg, 2-12 p.d.<br>Fentanyl tabl. Subl 100 mcg, 6 p.d.<br>Paracetamol daily<br>Pregabalin caps 2 x 75 mg 2 p.d.	Naproxen 250 mg now and then<br>Paracetamol daily
11	Pregabalin 50 mg 2 p.d<br>Pregabalin 300 mg 1 p.d<br>Amitriptyline 25 mg 3 p.d<br>Morphine 10 mg 5 p.d<br>Fentanyl plaster 12 mcg	Morphine 10 mg p.d.<br>Pregabalin 75 mg 3 p.d.<br>Amitriptyline 50 mg
12	Tramadol 50mg 2 p/d<br>now and then paracetamol/ibuprofen	No pain medication
13	Oxycodone 3 x 15 mg p.d<br>Nortriptyline 10mg<br>Paracetamol	Nortriptyline 10 mg<br>Incidental Paracetamol
14	Tramadol 50 mg 1-3 p.d.<br>Incidental paracetamol 1000 mg	Incidental paracetamol 1000 mg
15	Oxycodone c.r. 20 mg 5 dd	Sometimes Tramadol because of recent diagnosed herniated disc
16	Morphine c.r. 30 mg 2 p.d.<br>Morphine e.r. 10 mg 4 p.d.	No pain medication
17	Morphine 10 mg 2 p.d<br>Amitriptyline 25mg p.d.	Morphine 10 mg 2 p.d.
18	Tramadol drops 4 x 2d	No pain medication
19*	Oxycodone tablet 10 mg 5 p.d.<br>Oxycodone tablet c.r. 10mg 2 p.d.<br>Naproxen tablet 500mg 1 p.d.<br>Paracetamol 500mg 4 p.d.	Methadone 4 mg.
20	Lidocaine injections 20 mg/ml (HCL)<br>AMP 10 ml<br>Tramadol caps 50 mg 4 p.d.	No pain medication

21	Oxycodone c.r. 5mg 2 p.d.<br>Paracetamol 100 mg 3 p.d.	No pain medication
22	Oxycodone (c.r.) 30 mg 3 p.d.	Oxycodone 5 mg 1 p.d.
23	Gabapentin 300 mg 6 p.d.<br>Pregabalin 75mg 5 p.d.<br>Oxycodone c.r. 1 x 10 mg<br>Oxycodone e.r. now and then	Gabapentin 300mg 6 p.d.<br>Nortriptyline 25 mg 1 p.d.
24*	Oxycodone c.r. 30 mg 2 p.d.	Oxycodone 30 mg p.d.
25	Oxycodone c.r. 4 x 60 mg 4 p.d.<br>Oxycodone i.r. 5 mg 3 p.d.<br>Diazepam 20 mg 1 p.d.<br>Pregabalin 150 mg 2 p.d.<br>Omeprazole20 mg 1 p.d.	No pain medication
26	Tramadol 100 mg 2 p.d.	No pain medication
27	Tramadol HCL/Paracetamol 37,5/325 mg / 90, 1-3 p.d.	No pain medication
28	Oxycodone c.r. 5 mg 1 p.d.<br>Codeine 20 mg now and then	No pain medication
29	Amitriptyline 10 mg<br>Tramadol 50 mg 2 p.d.<br>Paracetamol 500 mg 8 p.d.	Amitriptyline 10 mg

p.d. = per day, i.r. immediate release, c.r. controlled release
